# Healthcare workers use of psychological support resources during COVID-19; a mixed methods approach utilising Pillar Integration Analysis

**DOI:** 10.1371/journal.pone.0267458

**Published:** 2022-04-27

**Authors:** Helen L. Richards, Joseph Eustace, Amanda O’ Dwyer, Andrew Wormald, Yvonne Curtin, Dónal G. Fortune

**Affiliations:** 1 Department of Clinical Psychology, Mercy University Hospital, Cork, Ireland; 2 Department of Psychology, University of Limerick, Limerick, Ireland; 3 Department of Nephrology, Cork University Hospital and HRB Clinical Research Facility at University College Cork, Cork, Ireland; Universitat Oberta de Catalunya, SPAIN

## Abstract

**Objectives:**

We sought to examine healthcare workers (HCWs) utilisation of formal and informal psychological support resources in the workplace during the first and third waves of the COVID-19 pandemic in Ireland.

**Methods:**

A convergent mixed methods approach was undertaken. Four hundred and thirty HCWs in the Mid West and South of Ireland responded to an online survey in terms of their use of psychological support resources during Wave 1 (April/May 2020) of COVID-19. Thirty-nine HCWs undertook in depth interviews at Wave 3 (January/February 2021), and a further quantitative survey was distributed and completed by 278 HCWs at this time. Quantitative data arising at Wave 1 and Wave 3, were synthesised with Qualitative data collected at Wave 3. A Pillar Integration Process (PIP) was utilised in the analysis of the quantitative and qualitative data.

**Results:**

Five pillars were identified from the integration of results. These were: a) the primacy of peer support, b) the importance of psychologically informed management, c) a need to develop the organisational well-being ethos, d) support for all HCWs, and e) HCWs ideas for developing the well-being path. These pillars encapsulated a strong emphasis on collegial support, an emphasis on the need to support managers, a questioning of the current supports provided within the healthcare organisations and critical reflections on what HCWs viewed as most helpful for their future support needs.

**Conclusions:**

HCWs who utilised supportive resources indicated ‘in house’ supports, primarily collegial resources, were the most frequently used and perceived as most helpful. While formal psychological supports were important, the mechanism by which such psychological support is made available, through utilising peer support structures and moving towards psychologically informed supervisors and workplaces is likely to be more sustainable and perceived more positively by HCWs.

## Introduction

Knowledge of the impact of the COVID-19 pandemic on the mental health and well-being of Health Care Workers (HCWs) is growing. A rapidly accumulating body of literature has illustrated significant levels of psychological distress with around 60% of HCWs experiencing common mental disorders [1], including anxiety [2–5], depression [3, 6], post trauma symptoms [1, 2, 7], perceptions of being stigmatised [8, 9], in addition to rates of sleep disturbance twice that reported in the general population [10]. Risk factors for psychological distress are complex and have been shown to include demographics (e.g. age [2, 11], and gender [2, 12]), career stage [11], and work related variables (e.g. frontline v non-frontline workers [2, 12]).

In line with the growing awareness of the substantial psychological burden of the pandemic on HCWs and the recognition of the need to support and protect both their physical and psychological well-being, there has been a number of rapidly deployed approaches aiming to support the psychological well-being of HCWs. These approaches include a wide range of strategies including telephone helplines, group based stress reduction activities and online distress management courses [13]; video support calls for HCWs [14]; digital learning packages [15]; in person and remote psychological / psychiatric support [16]; resilience interventions utilising peer and mental health support [17]; safe spaces for support and well-being [18]; peer support interventions [19]; expressive writing intervention [20]; mobile applications for screening for mental health difficulties [21]; psychological first aid (PFA) [15, 22, 23]; yoga and music therapy [24]; and mindfulness based interventions [25, 26]. Despite the range of studies on deployment of supports for HCWs, systematic reviews on approaches to support the well-being of HCWs note: insufficient quality of the research on staff support interventions to permit recommendations for healthcare organisations [27]; many interventional studies fail to report the efficacy of the intervention on HCWs [28–30]; interventions tend to use a multiplicity of approaches [30]; no study had utilised control groups and worryingly, very few had registered protocols [29]. These limitations indicate that the evidence for how best to support staff requires comprehensive elucidation.

When considering interventions for supporting the psychological well-being of HCWs, the preference of individuals is likely to be a key factor, as this will determine uptake and utilisation of services. Indeed it has been highlighted [13] that traditional offerings of professional services were generally deemed to be of little interest to HCWs in terms of trying to mitigate or prevent adverse mental health impacts of working during COVID-19. One recent study of ICU staff in Ireland showed staff believed that the availability of informal resources such as peer and departmental debriefs could be most helpful to staff during the pandemic [31], however this study [31] did not assess whether the participants had actually used such supports.

Given the energy that is currently directed towards appropriately supporting HCWs, it is essential to understand the supports utilised by HCWs during the pandemic and the perceived usefulness of these. To our knowledge, there is currently no published data that reports this information in relation to HCWs in Ireland. Through the use of a mixed methods approach that integrated quantitative and qualitative data, the current paper describes HCW utilisation of support resources in the first and third waves of the pandemic. The quantitative methods permit an examination of the uptake of supports used by different HCW professions and their satisfaction with each of the available supports, whilst the qualitative methods provide a complementary, yet richer understanding of the experiences of HCWs on the use and perceived usefulness of psychological supports. Via the integration of the quantitative and qualitative data, we sought to specifically answer the following research questions:

1. What psychological support resources do HCWs in the healthcare setting in the South/mid-West of Ireland report using? (Quantitative)
2. How do HCWs experience psychological support in the workplace setting? (Qualitative)
3. To what extent are the psychological support resources experienced as helpful by HCWs? (Mixed methods)
4. What recommendations can be made for future well-being resources for HCWs? (Mixed methods)

## Methods

### Design

A convergent mixed methods approach [32, 33] was undertaken across Wave 1 (April/ May 2020) and Wave 3 (January / February 2021) of the COVID-19 pandemic in Ireland.

**Fig 1:**
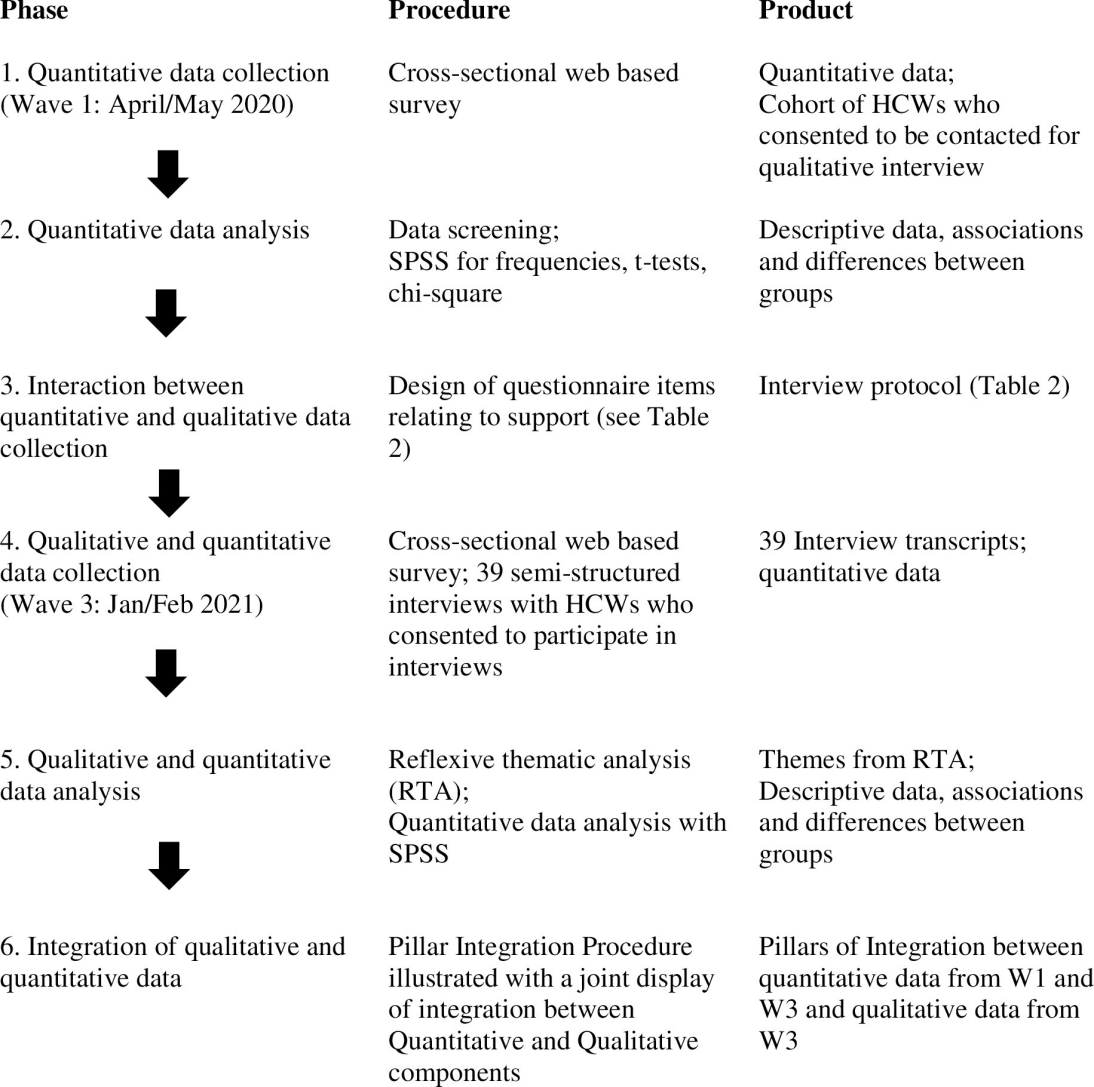
Phases and integration of approaches within the study. Adapted from Ivankova and colleagues [35].

An interactive approach was taken whereby analysis of the quantitative component of the study at Wave 1 contributed to the design of the qualitative semi-structured interview schedule used at Wave 3 [33]. Quantitative data was again collected at Wave 3, and the analysis of the quantitative data at Waves 1 and 3, alongside the qualitative data from wave 3 permitted an integrative interpretation of the results utilising a Pillar Integration Process [34]. This approach allowed a more nuanced and integrated examination of HCWs use of support resources. The procedural components of the study are outlined in Fig 1.

### Participants

HCWs were eligible to participate if they were employed within the hospital or community sites in the Mid West and South of Ireland. All occupational groups and ancillary services were eligible to participate. Four hundred and thirty HCWs were recruited during wave 1 of the pandemic and 278 HCWs were recruited during wave 3 from acute hospitals and adjacent community sites. HCWs were emailed from centralised hospital email servers and notices were placed on internal hospital intranet sites and notice boards to encourage participation by those who may not have had direct access to emails. Recruitment took place in April/May 2020 (Wave 1) and December 2020/January & February 2021 (Wave 3). Of 127 individuals who consented to be contacted for qualitative interviews, 39 responded to invitations to take part and were interviewed either via zoom or telephone during the third wave of COVID-19.

Ethical approval was granted by the Clinical Research Ethics Committee of the Cork Teaching Hospitals and HSE Limerick Hospitals ethics committee (CREC: ECM 4 (a) 09/04/2020 & ECM 3 (u) 09/04/2020 and UHL REC 041/2020). Local site approval was obtained for each of the individual hospitals and community health areas participating in the study.

### Demographics

Participants were asked to provide information on their gender, age, occupational setting (acute hospital, primary care/community, prefer not to say), profession (medicine and nursing, Health and Social Care Professionals (HSCP), administration, management, or support services), and the number of years working in that role.

### Measures

The research team generated a list of the available supports that could be accessed by HCWs. Participants were asked to respond as to whether: 1) they had utilised occupational health, employee assistance programme (EAP), work buddy system, end of shift huddles, psychological first aid (PFA), psychological support services, and up to three other supports in a free text format, and 2) how helpful they found the service they had accessed, by responding on a 10cm visual analogue scale from zero (not at all helpful) to ten (extremely helpful).

#### Interview schedule

Analysis of the quantitative component of the study at Wave 1 contributed to the design of the qualitative semi-structured interview questions about supports at Wave 3. Table 1 illustrates the survey item responses, and how they relate to the interview questions used.

**Table 1:** Interview questions and probes arising from survey item responses.

Responses to survey items	Interview protocol questions/probes
A low uptake by staff in relation to support resources offered.	Please tell me about your experience of working during the COVID-19 outbreak. • what helped you cope? • what made it more difficult? • What kind of support did you receive? Did you feel supported during the pandemic?
There were differences between occupational groups and their use of support resources.	What was the difference between providing care/work due to the pandemic and your usual care/work? (redeployment/managing different staff etc.) • how did you feel accepting the task? • what challenges did you encounter? What kind of support did you receive? • what kind of support would have been helpful?
Significant association between working in acute settings and use of formal resources.	
Over half of managers accessed support resources.	
Occupational health services were used most frequently and were rated as most helpful	In the context of your own health • what was the impact on your mental and physical wellbeing? • what support would be helpful?
A wide range of support measures were identified in free text responses, both internal and external to the workplace.	In relation to the kind of support you received outside of the work environment: • can you tell me a bit about these supports? Was there ever any conflict between these different sources of support?
Peer support was the most used and highest rated free text response.	
	Were your relationships impacted over this time? • would you have liked support with this? How? Who? • how did you manage this?
Of those staff that accessed support almost one third used more than one support resource.	Which of these supports you have mentioned do you feel are your main support system? • Has this changed since the pandemic? • How?
The majority of staff did not use any supports; what do HCWs think could be done about this?	Is there any advice you would like to give to improve the care of staff working in similar circumstances?

### Analysis

#### Quantitative analysis

IBM’s Statistical Package for the Social Sciences (SPSS) version 26 was utilised in the statistical analyses. Quantitative data were explored to examine for normality of distribution of continuous variables. Levene’s test was used to examine homogeneity of variance between groups. Based on the non-significance of these examinations, parametric tests were utilised throughout the analyses except where specifically stated. Descriptive statistics were used to describe the characteristics of the sample (e.g. gender, age, occupational category). T-tests permitted examination of differences between groups (e.g. HCWs who did and did not utilise support services, HCWs in a community or acute setting, and age or years qualified and use of support resources). Chi^2^ or Fishers Exact test as appropriate were utilised to examine the association between categorical variables e.g. occupational group and utilisation of support resources and Mann-Whitney U was utilised for differences in ratings of helpfulness of services over time.

#### Qualitative analysis

A reflexive thematic analysis was undertaken of the interview data utilising approaches described by Braun and Clark [36]. By exploring patterns of meaning between participants across the interview data we were able to explore the participants experiences related to the use of psychological support resources. Interview transcripts were transcribed verbatim by AOD and YC and quality checks undertaken by AOD, YC and HLR by checking the transcriptions against the recordings. This permitted initial familiarisation with the data. Sections of the interview data that were relevant to the research questions were identified by HLR and imported into NVivo. Codes were assigned to the interview data by HLR through a process of moving backwards and forwards throughout the transcripts, refining the codes as necessary. Once all the data had been coded, the codes were discussed within the research team and the codes were organised by HLR into possible themes which was based on the relationship between codes. An inductive approach was taken allowing the data to determine the themes. Each theme was checked for internal consistency, considering whether the data within the theme was coherent and homogeneous. Themes were checked against each other to identify whether there were clear distinctions between themes. Themes were named based on the meaning of the theme, and described using quotations to support the analysis.

#### Reflexive stance

Given the subjective nature of reflexive thematic analysis it is recognised that the coders subjective lens will have influenced the decisions made with respect to coding, analysis and interpretation. The research team comprised of clinicians and academics from a range of backgrounds and levels of experience. HLR, AOD, AW, YC and DGF all had experience of previously conducting qualitative analysis. HLR who coded the manuscripts and sorted these codes into themes is a clinical psychologist with over 25 years experience working in acute teaching hospitals in both the UK and Ireland.

#### Integrative analysis

A Pillar Integration Process (PIP) technique [34] was used to integrate the qualitative and quantitative data and present both the quantitative and qualitative data in a joint display [33]. By adopting the PIP we aimed to reduce any potential observer bias and to enhance the opportunities for the integration and synthesis of data, from both a visual and methodological perspective [34].

The PIP process was followed as per the originators guidelines [34], utilising four key stages. The listing of the raw data relating to the quantitative arm of the study was undertaken. Column A (Table 4) reflected the quantitative data from the survey, e.g. percentages of individuals who reported utilising each support, and column B the higher order categories manifested from the data in column A. Data matching from the qualitative arm of the study which reflected the content of the quantitative data was listed in column E in the form of quotations from the participants and the categories or themes from the codes was reflected in column D. This procedure allowed us to organise the data coherently and establish any patterns or data that did not link together. We then undertook a process of checking the data to ensure that all data had been included and the quantitative and qualitative rows were examined to see how well they matched or fitted together, before embarking on the integration of the results. The Pillar building (column C) emerged from the assimilation of the data drawing on themes, understanding and explanations which allowed us to develop a narrative of the data.

## Results

### Summary of quantitative results

At wave 1, of 513 HCWs who consented to take part and provided demographic information, 430 (83.8%) provided details on the support services they had used. No gender (X^2^ = 0.40, df 1, p = 0.53) or age differences (t = 0.77, df 437, p = 0.44) were identified between those who did and did not complete support-related data. At wave 3, 443 HCWs consented to participate and of these 278 (63%) provided demographic data and data related to their use of support services. Table 2 below indicates demographic and occupational related data for the HCWs Wave 1 and Wave 3.

**Table 2:** Demographic and occupational variables of the sample.

Variable	Value	
	Wave 1 (n = 430)	Wave 3 (n = 278)
*Gender* n (%)		
Male	57 (13.3)	44 (15.8)
Female	373 (86.7)	234 (84.2)
Age mean (SD)	42.82 (9.95)	43.02 (10.18)
Occupational setting n (%)		
Acute hospital	233 (54.2)	230 (82.7)
Community / Primary care	145 (33.7)	48 (17.3)
Prefer not to say	52 (12.1)	0
Occupational group n (%)		
Medical / nursing	148 (34.4)	102 (36.7)
Health and Social Care Professionals (HSCP)	138 (32.1)	71 (25.5)
Administration	81 (18.8)	53 (19.1)
Managerial	35 (8.1)	24 (8.6)
Support services (portering/ HCA/ catering)	26 (6.1)	28 (10.1)
Did not state a profession	2 (0.5)	0
Years qualified / in role mean (SD) range	13.77 (9.51) 0–41 years	14.22 (10.5) 0–40 years

Thirty-eight percent of HCWs at wave 1 and 26% at wave 3 responded that they used one or more support resource. Of those using support resources, at wave 1, 30.5% (n = 50) and at wave 3, 29% (n = 21) used more than one resource. There was no association between use of support resources and gender (X^2^ = 0.05, df 1, p = 0.83) and no significant age differences between those who did and did not report using support resources at wave 1 or wave 3 (t’s<1.63, p’s>0.05).

At both waves 1 and 3, HCWs who used support resources had been qualified/worked in their roles for the same amount of time as those who did not use support resources (t’s <0.61, p’s >0.54). There was an association between working in an acute setting and use of support resources (X^2^’s >6.17, p’s<0.05), with acute hospital HCWs utilising supports more than community services across both waves sampled.

At wave 1 of COVID-19, medical and nursing staff were more likely to access support resources than HSPC staff (X^2^ = 4.2, df = 1, p = 0.04), administration staff (X^2^ = 5.8, df = 1, p = 0.02) and support staff (X^2^ = 4.73, df = 1, p = 0.03). Additionally managers were more likely to access support than administration staff (X^2^ = 5.03, df = 1, p = 0.03) or support services (X^2^ = 5.03, df = 1, p = 0.03). At wave 3, the number of HCWs using any support resources listed had decreased. Medical and nursing HCWs used support resources significantly more in wave 3 than HSCP (X^2^ = 3.91, df = 1, p = 0.048) and management grades more than support services grades (p = 0.006). There were no other significant differences between occupational groups. Table 3 illustrates the proportion of workers from each occupational category accessing support resources.

**Table 3:** HCWs self-reported use of support resources at Wave one (n = 430) and Wave three (n = 278).

Support resource	Wave 1 (n = 430)		Wave 3 (n = 278)	
	Times used (%)*	How helpful was the service (median & range)	Times used (%)*	How helpful was the service (median & range)
Occupational Health	69 (35.57)	6 (0–10)	50 (18)	7.5(0–10)
Employee assistance programme	11 (5.67)	5 (0–10)	6 (2.2)	3.5 (1–10)
Buddy system	37 (19.07)	8 (0–10)	10 (3.6)	8 (5–10)
End of shift huddles	23 (11.86)	6 (5–6)	16 (5.8)	7 (4–10)
Psychological First Aid	12 (6.19)	4 (0–10)	1 (0.4)	4 (4)
Psychological support services	12 (6.19)	5 (0–10)	2 (0.7)	7 (4–10)
Other resources (e.g. collegial support, chaplaincy services, private counselling, coaching, supervision, HSE stress control)	30 (15.46)	8 (0–10)	10 (3.6)	8.5 (3–10)

* % illustrated is number of respondents who used the service at least once.

Of the other individual idiosyncratic support resources that were utilised at Wave 1, resources internal to organisations were most frequently used, with peer support the most popular. In terms of the helpfulness of resources accessed, the buddy system and individually selected resources were rated by staff as being the most useful (median = 8), with the more formal professional approaches of PFA, psychological support services and EAP reported by participants to be the least helpful (median scores ≤5). At wave 3, additional idiosyncratic responses included the use of counselling (n = 5) and using GP’s (n = 3) for support.

Overall more staff used support services at Wave 1 than Wave 3 of COVID-19 in Ireland (X^2^ = 10.58, df 1, p = 0.001). Given that psychological support services and psychological first aid were used very infrequently (<5 times at Wave 3) it was not considered viable to subject these supports to further statistical scrutiny. The buddy system was associated with increased use at Wave 1 compared to Wave 3 (X^2^ = 6.91, df 1, p<0.01), but no other associations between the Waves of COVID-19 and use of particular support services were identified. In terms of perceived helpfulness of services, occupational health was the only service to be rated differently between the two waves, receiving higher ratings at Wave 3 (U = 1770, p = 0.002). There were no significant associations between occupational groups use of support services at Wave 1 and Wave 3 (X^2^ = 7.88, p = 0.10) and no associations between any occupational group and their use of particular support services between Wave 1 and 3 (X^2^ <6.91, p’s>0.05). Both medical and nursing (U = 366, p = 0.02) and administration staff (U = 29, p = 0.03) rated occupational health services as more helpful at Wave 3.

### Summary of qualitative results

A total of 127 HCWs indicated they would be willing to take part in qualitative interviews, with 39 formally consenting to participate in the interview process. Twenty-nine were female and 10 male, with 25 working in acute settings, 12 in the community and 2 HCWs working across both settings. Nine of the HCWs were redeployed during the pandemic to swabbing or call centres. Due to concerns around anonymity expressed by many of the interviewees, occupations were grouped using categories utilised in the quantitative arm of the study, and all occupational groups were again represented: medical and nursing (M&N; n = 10), Health and Social Care Professionals (HSCP; n = 15), administration (Admin; n = 7), managerial (n = 5) and support services (SS; n = 2).

Reflexive thematic analysis was undertaken on the 39 interview transcripts. In efforts to be succinct, and in line with previous mixed methods studies [37] themes are described within the integrated analysis as outlined below. Analysis of the data arising in the interviews identified four themes from the data relating to psychological / well-being support for HCWs in the workplace. These were, “my colleagues got me through this”, “psychologically minded management”, “the smoke and mirrors of staff-support” and “where do we go from here?”.

### Integration of qualitative and quantitative findings

The PIP process resulted in the emergence of five pillars from the integrated qualitative and quantitative results. These were, a) the primacy of peer support, b) the importance of psychologically informed management, c) a need to develop the organisational well-being ethos, d) support for all HCWs, and e) HCWs ideas for developing the well-being path. Table 4 illustrates the integration of the findings and the resultant pillars. Each of the pillars are reported below.

**Table 4:** A joint display of the connections between the quantitative and qualitative data arising from the study.

Survey findings		PILLAR	Interview findings	
A	B	C	D	E
19% used buddy system, most helpful, rated 8/10	Data illustrating that collegial supports used most frequently and rated higher than more formal psychological supports	Primacy of peer support	Being there for your colleagues	“…everyone was just kind of, was there for each other and you know, kind of, kind of egging each other along, you know” (P22, manager, acute)
12% used end of shift huddles				“We ended up within the team supporting each other” (P33, HSCP, acute)
				“I think the support from colleagues was great. Just to feel out how everybody else was doing. Everybody is kind of feeling the same and that was supportive” (P14, HSCP, community)
			Loss of peer support with redeployment	“…That’s been that’s probably been the most disheartening thing about it I would say…so, not so much management side of things but our own colleagues.” (P25, HSCP, community)
				“…some of the people who did the swabbing were kind of getting pushback from the people who didn’t… “You’re going to be bringing COVID in here”, and so there was a little bit of tension” (P19, HSCP, community)
				“…our management was redeployed to another role. So, we were left with no management to help us along and we were quite fragmented as it is…” (P14, HSCP, community)
			Peer support alone not sufficient enough	“…I do, find now a year on, we’re kind of not enough for each other…it’s not that were sick of each other, but it’s like ok we’re all exhausted now, we’re all needing a break.” (P34, M&N, acute)
				“…healthcare workers are gonna be dropping like flies and it won’t be because they’re getting COVID. It’ll be because they’re absolutely exhausted and burnt out. And you can’t vaccinate people against that.” (P15, HSCP, community)
				“I think people in Ireland just really rally around one another in times. But I think we’re getting tired….you know; I think some of us are really tired.” (P1, Admin, acute)
This data source reflects the interview data only		The importance of psychologically informed management	An approachable line manager	“…being able to talk to my boss or (), my bosses boss about how I was doing and stuff am it did help. And I think you know… I knew I could always go to her for support.” (P17, admin, acute)
				“My line manager is very, very supportive…so when there were times that I was feeling pressured, I always knew I could go to her. . .” (P24, M&N, acute)
				“I suppose being able to chat with my manager, and knowing that I could, so she kept in regular contact and that was really good” (P19, HSCP, community)
				“I would have been telling our, my own colleagues about like employee assist, if you know feeling overwhelmed or come to us.” (P16, management, acute)
			Perception of poor management support	“…I don’t feel supported from, from the top down I suppose, that would be my, my take on it. . .They just don’t seem to be seeing the fact that their staff are under severe pressure and stressed you know.” (P29, M&N, Acute)
				“I think a lot of people need leadership skills. They might have management skills but they don’t have leadership skills, COVID and non-COVID times.” (P34, M&N, acute)
			Invisibility of management	“I appreciate that people are exhausted. I appreciate that those leaders are exhausted but they also need to… they’re invisible now people don’t see them.” (P33, HSCP, acute)
				“I just think leadership, support, gratitude, all those things just needs to be upped from… I can only speak about our organization so you know. That’s, that’s, it’s blatantly absent now” (P34, M&N, Acute)
				“I think initially like you know the visibility of Senior management probably was a disgrace and you know social distancing and all that was used as a kind of thing, so I think visibility from the senior team. Better communication, listening to staff…I think the most important thing for something like this is that managers and leaders are out there showing that support. I think you’ve got that sometimes I think, if that support is shown. And yes, it won’t change anything that’s going on. Sometimes that’s all you need to know.” (P6, Manager, acute)
			Knowing how to help	“…just to listen to the venting as opposed to try to jump in and go oh, I can do this this, this and this to help…. You don’t necessarily need to go fix it or do anything about it, but that just someone has gone yeah that was a shite day but you did great and you did all you could.” (P30, Management, acute)
				“…there’s very little supervisor management courses. Or communication courses available to help you to develop your personal interpersonal skills, you know.” (P23, HSCP, acute)
62% and 74% did not use any support resources at wave 1 and wave 3 respectively.	Low uptake of available supports	A need to develop the organisational well-being ethos	Smoke and mirrors	“And there is that sense, I suppose. I have and I know that my colleagues have it as well, but the system isn’t gonna mind us, so really we have to take responsibility for minding ourselves. There isn’t we…. unfortunately…. and that’s kind of sad in a way.” (P15, HSCP, community)
				“… in Ireland sometimes we don’t have all of the structures in place to sort of look after you formally. . .am informally there are all sorts of things…” (P11, M&N, acute)
				“There’s a blind eye thrown I think if they see someone distressed or whatever that they kind of look away they don’t want to know about it.” (P29, M&N, acute)
				“I would like to … have the health and wellbeing, you know, filtered down to the staff. I would, I would like to see, you know, the very basic, especially in the hospitals. I would like to see the basic of, you know, manners in respect and value being incorporated. Not just on a billboard. But actually, filtered down to the staff on the ground level, of all, of all levels; it doesn’t matter what level you’re on. And you know, that kind of acknowledgment and value.” (P33, HSPC, acute)
At wave 1:<6% used EAP; <7% used psychological support services;<7% PFA.	Data illustrating low uptake of psychological supports within workplace		Poor knowledge of supports	“And you know, we’re all, we’re always reassuring ourselves that, you know, we are part of a team. We’re trying to be open and voice our concerns…am but am it’s, it’s not like anyone offered us any counselling or guidelines.” (P10, SS, acute)
				“…I kind of thought that maybe the HSE should have had a helpline, as in if you were really struggling you contact this helpline…” (P5, Admin, acute)
At wave 3:<3% EAP;<1% psychology support services; <1%PFA				
			Ineffective communication systems about supports available	“There was a lot of push with the psychological services being available….But nursing staff are never on email. They’re never able to log in…..They don’t have the time. They’ve to share workstations.” (P31, Admin, Acute)
				“I wasn’t provided with any information on employee assist…. programme that was open to me… yeah there was nothing done.” (P29, M&N, acute)
			Concerns with usability and confidentiality of Employee Assistance Programme (EAP)	“I think the confidentiality thing, I dunno maybe I sound paranoid, but like, I just there’s, there’s a great fear over that.” (P23, HSCP, acute)
				“People didn’t feel that they could just phone them [EAP] up to talk about having a bad day…which many people were having at the time.” (P35, HSCP, Acute)
				“EAP (Employee Assistance Programme) is gone off site…I think some of it you book online or you can talk on the phone so I think that just puts people off to be honest. When it was onsite I think they were more inclined to use it…but it’s gone off site I think they just don’t bother” (P6, manager, acute)
			“Not ok to not be ok”	“it was really like more of an attack when I got the first phone call. After telling this person my issues…. Um, so I suppose if they understood, and if they had taken the time to sit down and understand what could work. If they had asked me if there was anything that they could do. It was really just the opposite. It was well what are we going to do with you now.” (P1, Admin, Acute)
				“….It’s a case of get on with it, do what you do, and you know, who, who, who wants to be looking after your mental health like you know…()…There’s a blind eye thrown. I think if they [management] see someone distressed or whatever that they kind of look away, they don’t want to know about it.” (P29, M&N, acute)
				“…there’s parts of the HSE [healthcare organisation] that seem to have a rigid management structures in place where to actually admit you’re vulnerable and you’re struggling is tantamount to saying you’re not doing your job well and that you shouldn’t be here.” (P19, HSCP, community)
Wave 1 84% completed survey	Uptake of offer to participate in survey and/or interview		Time as a barrier	“I felt that I couldn’t be taking more time out to maybe go on a call at 5:00 o’clock in the day to say OK, this is how I feel about today or this is what I think about today and you will not have that my time is precious” (P5, Admin, acute)
Wave 3 63% completed survey				
127 HCWs agreed to take part in interviews				“I know we were offered the am, the Employee Assistance Program, but you know, I just didn’t have time.” (P7, HSPC, acute)
39 responded to invitation and participated (31%)				
			Tokenistic nature of supports	“So you’re expected to give up your free time to attend something which is actually meant to support you with your work, so it’s not so it doesn’t ever feel to me that it’s sufficiently valued that you could actually be, you know, allowed to take time out of your working day to attend to your own physical and mental health needs apart from obviously the lunch time Pilates or exercise classes or whatever they were doing so. It is a bit of a contradiction for me in that” (P15, HSCP, community)
				“….they would always say, “Now, I would like this minuted”, and be sure to tell you that there is the staff line support if you want help, and there is the whatever other listing of support they would call out and, “make sure that’s minuted.” (P33, HSCP, acute).
				“Have you thought about they don’t have a workstation, or access or time or…so, that would be, yeah, that would be the main thing because they’re both linked to health and wellbeing…” (P31, Admin, Acute)
Managers used supports more than administration and support services	Who accessed supports? All ages, occupations, across all levels of experience. No impact of gender	Support for all Healthcare workers	Lack of supports for management	“They got the employee assistance programme, they got a person in to speak to all the managers. . . and I thought that was great and we all went to it, and focus was very much about which is important, the focus was, how do you deal? How do you as managers here deal with the staff underneath here in your remit and how to mind them in a time of COVID with their questions and their queries and their unknowns etcetera, which was fine. But I was, we were a lot of us as managers were sitting there going well we as managers need help and support as well.” (P34, Management, acute)
51.4% managers accessed supports				
				“…we see how stressed she [manager] is and no one is recognizing from any level, how, what she is doing..()… she said I need you to not be kind to me ’cause she said I’ll break…()…she said I’m not going any deeper because I need a game face and I need to keep going.” (P24, M&N, acute)
			Impact of poor support for managers on staff	“We were just fire-fighting the situation the whole time. So, when I would look for support or express, like, you know, the stress around—and it’s like, “I’m in the same boat as you.” And like, yeah, that’s fine, but, “How do I manage it? Or can I manage it? Do you’ve any recommendations?” (P31, Admin, acute)
				“by the July, we were literally burnt out and we kind of said to our manager, “We are burnt out we are literally burnt out” ….they gave us a slip of paper and said, “Here is the Employee support line. Ring it. Everybody’s burnt out. Just do what you’re paid to do.” (P33, HSCP, acute)
Acute settings used support resources significantly more than community (X^2^’s >6.17, p’s<0.05)	Use of support resources across settings		Inconsistencies across settings	“I was extremely disappointed at the support from the hospital. Um they were very disorganised. Um very little support. . . .” (P33, HSCP, acute)
				“we were encouraged from the beginning there was a pandemic to link with our colleagues more. There were small groups assigned with kind of a leader in the group…who was to link with other members of that group on a regular basis….” (P14, HSCP, community)
				“I think like I’ve heard about some people having a buddy system …I think that would have been very useful.” (P3, M&N, community)
Identification of a wide range of idiosyncratic supports utilised by 15% of HCWs in survey (wave 1).		HCWs ideas for developing the well-being path	How do we move on?	“I think we were saying among ourselves, that we need probably more support to, to manage the frustration that has been emerging among the staff, you know? About how to, how we move on from here and how do we do it?”(P14, HSCP, community)
				“You will see you can contact the EAP here and you could do this here. I don’t know if that’s enough really.” (P6, Management, acute)
			A suite of support measures	“I think just maybe having maybe like a check in service or something that …just to see how they’re doing.” (P3, HSPC, community)
				“Maybe a consultant or head nurse to give a little briefing once every few weeks, you know, check in with them in a room in a group setting. That might be good…” (P32, Admin, acute)
				“…I like to meditate…()…if that kind of facility maybe would be available somewhere around the hospital on the hospital grounds. (P10, SS, acute)
				“…it’s therapeutic actually just talking to somebody else…()…sometimes it’s just that you need somebody that’s not a friend to have a chat with or a debrief with” (P28, SS, acute)
			What’s helpful?	“…back through the years we would always say: “Oh the HSE couldn’t give a shit about us” or whatever …but actually, there’s been so much stuff around kind of mindfulness courses, and you know, stress am… stress relieving ideas kind of thing.” (P25, HSCP, community).
				“I think it was things like I don’t know like daily, what do they call them, daily staff moments or something coming to your email. I didn’t need them but I think they were helpful and they would be good if someone was struggling.” (P10, SS, acute)
				“…we do get emails about the EAP and counselling I haven’t joined it here but I did use it in the last job, I would say that it’s brilliant. It’s like you know I, I just think it’s a, it’s great to know that that is there in the background…. If you need it.” (P4, HSCP, community).

#### Pillar one—primacy of peer support

The findings from both the quantitative and qualitative arm of the study converged in relation to participants’ use of collegial peer support mechanisms as a primary means of support while working through the pandemic. Overwhelmingly HCWs referenced their work colleagues, in particular peers or members of their team, as the principal point of support through the pandemic. This was echoed in the survey in that the most used supports were reported to include occupational health and other collegial supports, e.g. buddy system and end of shift huddles, in contrast to the more formal approaches of EAP and PFA. The majority of HCWs viewed peer support as an informal and reciprocal approach, in that support was both given and received by staff. There was an underlying sense of relief that HCWs could share their experiences with their peers. There was some recognition that despite the value and importance of peer support, the impact of it on colleagues well-being might be reducing. Particular difficulties were highlighted for individuals who were redeployed. While there was a recognition of good ‘team spirit’ for redeployed HCW, this went hand in hand with the perception that colleagues from their pre-pandemic work places were not always supportive. This was related to a concern that redeployed HCWs were contributing to a backlog of work for colleagues ‘left behind’, and when working across roles there was a perception that they may add to the risk of COVID-19 being spread. In these scenarios HCWs identified disappointment at the loss of collegiality.

#### Pillar two- the importance of psychologically informed management

The importance of having psychologically informed management was explored in the interviews only. HCWs across all professional groups identified that having an approachable line manager was a key source of support and was spoken of very positively while working during the pandemic. There was also recognition from managers about the importance of supporting their staff. Conversely, when the management support was perceived as lacking, whether this be from a psychological or practical perspective, or at line management or corporate level, this was observed to have a detrimental impact on HCWs under their management. The visibility of management in relation to supporting staff was reported as being an important element in terms of HCWs perception of support, and “invisible management” was perceived as indicative of an unsupportive environment. Managers knowing how to support their staff whether that be via practical or psychological means, and in particular being able to listen without “trying to fix” was highlighted by HCWs as a key psychological resource from a support perspective.

#### Pillar three- a need to develop the organisational well-being ethos

The integration of data in relation to this theme encapsulated the idea that the system of support for HCWs had a number of weaknesses ranging from the more simple elements such as awareness of supports available to a perception of tokenistic provision of such support. HCWs reported a recognition that they needed to take actions to look after themselves rather than rely on supports within the system. This was further evidenced by the perception that staff who did express difficulties with work related stress and mental health difficulties were poorly managed. There was an observation from HCWs that “it’s not OK to not be OK” and of both felt and enacted stigma associated with expressing difficulties related to mental health in the workplace. When considering the EAP specifically, some HCWs expressed ambivalence about its usefulness. One of the most striking features arising from the data was that many HCWs were unaware of the EAP service available. HCWs also erroneously held the view that the EAP was reserved for HCWs working directly with COVID-19 patients. Confidentiality of the service was also a concern to some HCWs. As such the EAP was generally viewed as not a particularly accessible or useable source of support for the majority of HCWs. The low numbers of HCWs accessing the EAP may thus be contributed to by these apparent weaknesses in the knowledge held by HCWs about the EAP service.

Well-being supports were perceived as being tokenistic by HCWs who felt that managers and organisations were ‘covering their backs’ by informing staff about the service. There was a perception that by telling HCWs about the EAP permitted managers not to address challenges faced by HCWs in the workplace during COVID-19. The expectation that HCWs would avail of such supports in their own time rather than during working hours was reported to lead to HCWs not using the service and reinforced the perception of this support being tokenistic.

#### Pillar four -support for all HCWs

Data from the survey indicated that HCWs who accessed support services were not more likely to be male or female, young or old, experienced or junior. Indeed, within this pillar, there was recognition that the impact of the pandemic was being felt at all levels within the hierarchy of management within services and the organisation in which they existed. Managers roles were perceived as supporting others rather than being supported, and there was an observation of a missing piece within the supportive structures in place. This dichotomy of the manager as ‘supporter’ rather than ‘supported’ was highlighted by both managers themselves and employees in their charge, and consequently the detrimental impact of poor support for managers on the HCWs they managed was a shared concern. Alongside the support needs for managers this pillar also incorporated the perceived difference in experience between acute and community services. Significantly more HCWs in acute settings reported using formal supports during both waves. This aligned with a perception for some that a cascade of top down support within the organisational structure of the acute setting was more difficult to access, perhaps necessitating increased use of formal supports in this setting. Additionally there was recognition that some staff were able to access supports that others were not, and that this was dependent on innovations and leadership from within services, and some supports typically would not have been available to HCWs in community settings, such as end of shift huddles.

#### Pillar five–HCWs ideas for developing the well-being path

A wide range of idiosyncratic supports were identified by HCWs which matched with the data from the interviews. While highlighting what had been helpful resources, many staff recognised the limits of what was currently on offer, and offered ideas as to what they believed was required in order to adequately support HCWs in the workplace in the future. This ranged from the recognition that more cohesion, support, direction and guidance was needed from management in supporting staff, to the specifics to what was required. There was an overwhelming acknowledgment that while progress had been made in terms of supporting HCWs, there was much more that could be done from a practical and cultural perspective within the services and organisations in which staff worked. HCWs highlighted practices they had heard were engaged in by other services, they cited the need for supportive managers, regular debriefs and the availability of ‘helplines’, to quiet spaces within the work place where individuals could go to decompress, read or meditate. The range of supports identified was varied and reflected a stepped and non-prescriptive approach which may help to equip services and organisations to respond in a more cohesive, supportive, preventative and responsive way to HCWs on the continuum of support needs.

## Discussion

We present results of the first mixed methods study to investigate HCWs utilisation of support strategies in the first wave and third waves of the COVID-19 pandemic in Ireland. Five pillars were identified from the study that centred around: the primacy of peer support; the importance of psychologically informed management; a need to develop the organisational well-being ethos; support for all HCWs; and HCWs ideas for developing the well-being path.

The principal findings arising from the study highlighted the importance of the collegial support system within the workplace for HCWs during the pandemic. The significance of peer support and psychologically minded management for the participants in the current study are commensurate with recent studies in healthcare settings that have highlighted the importance of in-house peer support and psychological support from managers [31, 38, 39]. This is important as it has been suggested that interventions and resources for HCWs tend to focus on supporting HCWs once they are experiencing difficulties rather than building the organisations culture of supporting staff [40]. Encouraging camaraderie and ensuring that managers are approachable has been previously endorsed as potential ways to help support and minimise the deleterious psychological outcomes for HCWs working during infectious diseases outbreaks [38]. There is good evidence that when HCWs have a supportive supervisor, mental health is likely to be protected [41] while poor communication with supervisors is associated with burnout [42]. Moreover, there is evidence that relatively simple interventions to improve active listening skills increases managers’ confidence in recognising, speaking with and supporting staff with mental health challenges [43].

We found that the primacy of peer support impacted disproportionately on those who were redeployed to other roles outside their teams. Firstly, they were moved away from their usual networks of support and secondly in some cases they experienced some negative responses on retuning to pre-pandemic workplaces due to perceptions of increased workloads due to their absence. There is substantial research evidence which suggests that the environment in which a person finds themselves impacts the salience of their identity and the supports that they feel are available to them particularly during transitions. Across a range of social contexts, identity and support are shaped by the groups with which people identify and with whom they feel they belong, and affiliation with these groups may have a downside for staff when they are moved outside the group [44, 45].

The pillar of “support for all HCWs” highlighting the importance of support for all staff and all levels of management, is commensurate with the elements of peer support and psychologically minded management outlined above. Managers both expressed the need for support, and accessed support significantly more than support staff and administrators in the first wave of the pandemic. There is research suggesting that managers in the healthcare setting were at increased risk of experiencing psychological distress during the pandemic [7]. It has been highlighted in the current study and elsewhere [46] that managers at all levels are not exempt from experiencing stress and/or psychological distress. A system of more experienced managers looking out for more junior managers might be helpful and could be effectively operationalised within a peer support network.

The findings integrated within the Pillar “a need to develop the organisational well-being ethos” highlighted a number of issues around the provision of supports. There was perception of a poor uptake of more formal supports such as the EAP, and similar results have been reported in a recent study from the UK [47]. Our study however has added to this literature and has highlighted possible reasons why uptake might be considered low. It has also been noted that HCWs may not appropriately prioritise their own mental health [7], and existing formal support mechanisms such as EAP require HCWs to initiate contact with the resource. This requires an individual to recognise and act on their potential need. Furthermore, in some cases more formal psychological approaches may not be best positioned to respond to the problem when the key aspect of the challenge for HCWs may be organisational or systemic in nature, for example, concerns around PPE and infection [48], collegial or team difficulties, and work scheduling issues such as appropriate rest breaks [13].

One of the most emotive issues for HCWs interviewed was the expectation that support had to happen in their own personal time and is likely to contribute to the view that well-being is not important [49]. HCWs also reported reluctance to self-declare mental health difficulties, as they feared being perceived as unable to do their job; indeed some HCWs reported negative experiences when they did disclose such difficulties. Fears around disclosure of mental health difficulties and being perceived as not being ‘up to the job’ may also be perceived of as a potential barrier to accessing more formal supports within the workplace [50–52]. It is likely that a more consistent and direct message within organisations that recognises that everyone, regardless of grade or role, may experience distress and can actively seek supports might be helpful.

How we progress and build in terms of staff supports (Pillar 5) is a key concern of HCWs. We note that at this stage of the pandemic, there remain few good quality studies which report the best way to support HCWs [29, 30]. Our qualitative data outlined a wide range of resources which HCWs were utilising and this was endorsed in terms of identification of resources that HCWs believed would be helpful in the future. While a suite of potential supports were identified, perhaps reflecting the idea that a stepped approach to supporting HCWs will be required in line with their needs [53] it is notable that the majority of these revolved around ‘on the ground supports’ emerging from HCWs own peer networks. Many HCWs identified ‘checking in’ or the availability of ‘a chat’ as being potentially helpful reflecting the perceived value of proximal peer support for HCWs.

### Strengths and limitations

The study’s strengths include the mixed methods design across waves 1 and 3 of the pandemic and analytic technique implemented with cross-referencing of the results from the quantitative and qualitative components of the study (PIP). However, there are some limitations of the study. As participants self-selected, there may have been some bias in their responses. This limitation may be modified by the observation that a variety of occupations and management groups participated across both quantitative and qualitative streams of the study, adding to the value of this piece of work. Regarding the survey design, it is possible that the use of a free text responses format that asked about other supports utilised, may have resulted in some individuals not considering their use of informal resources such as peers as being relevant in the context of the survey. Nonetheless, the qualitative data collected identified a range of challenges associated with formal supports and it is suggested that these are addressed in order to optimise the potential for such services to more adequately support staff.

### Conclusions

The results of this mixed methods study of HCWs across waves 1 and 3 of the COVID-19 pandemic in Ireland suggest that it is the potentially simple collegial strategies, such as peer support and psychologically minded management, built up from within an organisation that may be perceived by HCWs as the most helpful to them during the pandemic cycle. Our interpretation utilising the PIP has provided helpful data to explain why individuals utilised certain support mechanisms and resources and has provided key data in relation to issues around perceived accessibility and potential obstacles to utilising these services. Moreover, the opportunity to build on the value of HCWs preferred first option for support, that is peer support, across all levels of the workforce is of importance based on the data in this paper. It is also likely that over time, some individual HCWs may experience mental health difficulties that require formal intervention and as such responding with evidence-based formal psychological support will be additionally required on that basis. It is reasonable to assume that as time passes, HCWs may become more adept at identifying what works best for them in terms of support mechanisms and other support resources including formal psychological intervention may become more important at this time [54]. It is likely that a suite of resources, focusing primarily on collegial approaches will be required to meet to the needs of HCWs and settling on one strategy as a means for organisations to feel that they discharged their duty to support HCWs is unlikely to be helpful.

## Supporting information

S1 File Interview questions and probes. (DOCX)
