# Supplementary material for: Healthcare workers use of psychological support resources during COVID-19; a mixed methods approach utilising Pillar Integration Analysis
Source: PLoS One. 2022 Apr 27;17(4):e0267458. doi: 10.1371/journal.pone.0267458 (PMC9045652; doi:10.1371/journal.pone.0267458)
Supplement: S1 File — (DOCX) [file pone.0267458.s001.docx]

**S1 File: Interview questions and probes**

| **Interview protocol questions/probes** |
| --- |
| Please tell me about your experience of working during the COVID-19 outbreak.   - what helped you cope? - what made it more difficult? - What kind of support did you receive?   Did you feel supported during the pandemic? |
| What was the difference between providing care/work due to the pandemic and your usual care/work? (redeployment/managing different staff etc.)   - how did you feel accepting the task? - what challenges did you encounter?   What kind of support did you receive?   - what kind of support would have been helpful? |
| In the context of your own health   - what was the impact on your mental and physical wellbeing? - what support would be helpful? |
| In relation to the kind of support you received outside of the work environment:   - can you tell me a bit about these supports?   Was there ever any conflict between these different sources of support?  Were your relationships impacted over this time?   - would you have liked support with this? How? Who? - how did you manage this? |
| Which of these supports you have mentioned do you feel are your main support system?   - Has this changed since the pandemic? - How? |
| Is there any advice you would like to give to improve the care of staff working in similar circumstances? |
